# Auricular Melanoma Excision: Excision of Auricular Melanoma

**DOI:** 10.1097/SAP.0000000000004501

**Published:** 2025-09-13

**Authors:** Hamish Thomson, Millie Chart, Shomari Zack-Williams, Christopher Jones

**Affiliations:** From the a School of Medicine, University of Liverpool, Liverpool; b The Mersey Regional Burns and Plastic Surgery Unit, Mersey and West Lancashire Teaching Hospitals NHS Trust, Knowsley, United Kingdom.

**Keywords:** melanoma, ear, cartilage-spared, cartilage-removed, excision

## Abstract

Preserving auricular cartilage can have satisfactory aesthetic outcomes and retain the functional integrity of the ear. Limited research exists on the oncological sequelae of preserving or removing cartilage when excising auricular melanoma. This systematic review compares outcomes between cartilage-sparing and cartilage-removing techniques in the treatment of auricular melanoma. A systematic literature search of PubMed, Scopus, and MEDLINE acquired 23 studies; following independent screening using a PICOT framework-guided inclusion and exclusion criteria and subsequent quality assessment, 6 retrospective studies were included in the final review. The literature suggests that there is a difference in recurrence rates and disease-specific mortality between cartilage-sparing and cartilage-removing excisions. Recurrence and disease-specific mortality appeared to be higher in studies that excised cartilage. However, these findings are inconclusive and undermined by several critical limitations. The explanation, direction, and magnitude of this difference remain unclear, and further long-term randomised control trials are required to confirm the differences in oncological outcome between cartilage-sparing and cartilage-removing excisions of auricular melanoma.

Auricular melanoma (AM) is an uncommon yet clinically significant malignancy of the external ear, representing up to 5% of all cutaneous melanoma cases, with a 5-year survival rate of ~74%.^1,2^ Data from 2006 reported that 1% of cutaneous melanomas were auricular in origin, suggesting a potential rise in the incidence over time.^3^ The mainstay of treatment for AM is surgical excision with histologically clear margins, in accordance with NICE guidelines.^4^ The auricle's lymphatic drainage is notably complex and variable; although the most common drainage basins include the level II cervical, preauricular, and postauricular nodes, sentinel lymphatic flow may extend to any ipsilateral cervical node (Fig. 1).^1,5^

**FIGURE 1:**
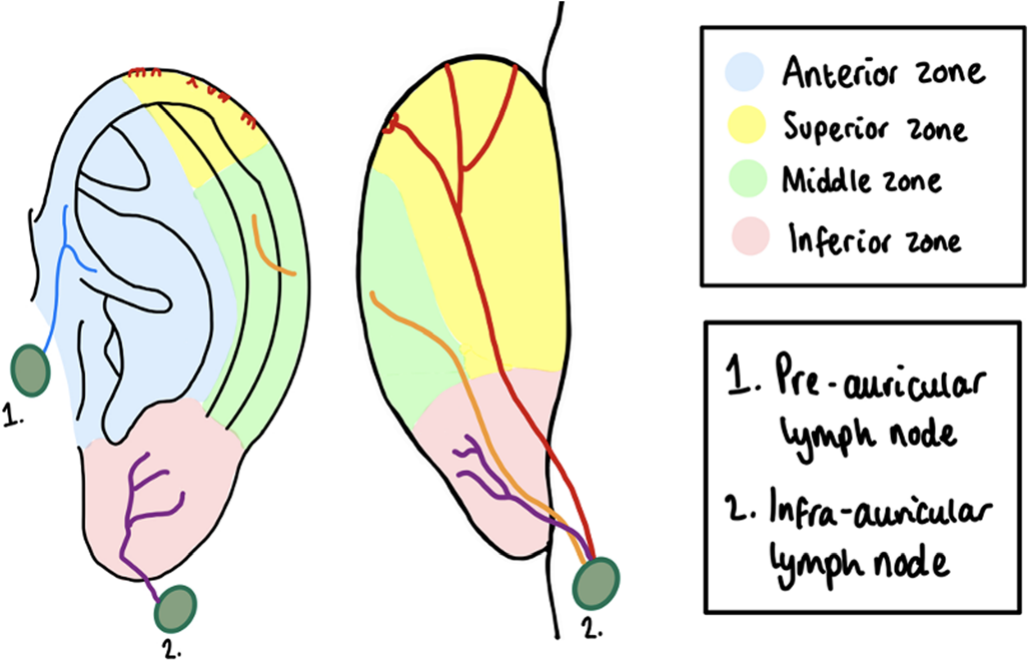
The auricular lymphatic system.

**TABLE 1:** PubMed Search Strategy

Date: March 20, 2025		
Number	Term	Results
1	(melanoma [tiab])	146,032
2	(cartilage-sparing [tiab]) OR (cartilage sparing [tiab]) OR (sparing of cartilage [tiab]) OR (cartilage-preserving [tiab]) OR (cartilage preserving [tiab]) OR (preservation of cartilage[tiab]) OR (cartilage preservation [tiab]) OR (cartilage-retaining [tiab]) OR (cartilage retaining [tiab]) OR (retainment of cartilage [tiab]) OR (cartilage-conserving [tiab]) OR (cartilage conserving [tiab]) OR (conservation of cartilage [tiab]) OR (cartilage-harvested [tiab]) OR (cartilage harvested [tiab]) OR (cartilage-harvesting [tiab]) OR (cartilage harvesting [tiab]) OR (harvesting cartilage [tiab]) OR (cartilage-excision [tiab]) OR (cartilage excision [tiab]) OR (excision of cartilage [tiab]) OR (cartilage-resection [tiab]) OR (cartilage resection [tiab]) OR (resection of cartilage [tiab]) OR (cartilage removal [tiab]) OR (removal of cartilage [tiab]) OR (chondrectomy [tiab]) OR (chondrotomy [tiab])	6589
3	(ear [tiab]) OR (auricular [tiab]) OR (auricle [tiab]) OR (pinna [tiab]) OR (external ear [tiab]) OR (middle ear [tiab]) OR (inner ear [tiab]) OR (helix [tiab]) OR (antihelix [tiab]) OR (lobule [tiab])	219,931
4	1 AND 2 AND 3	12

Achieving oncological control while preserving auricular form and function through cartilage-sparing excisions remains a challenge to surgeons. The auricle is composed of a single plate of avascular elastic cartilage perforated by small vascular channels.^6^ The decision to preserve or excise auricular cartilage alongside the melanoma is influenced by multiple clinical and histopathological factors, including Breslow thickness, presence of ulceration, tumor location, and required excisional margins. These variables guide the extent of resection necessary to balance oncological safety with preservation of auricular structure. However, there is limited evidence regarding the difference in oncological sequelae between cartilage-sparing and cartilage-preserving excisions.^7^

More radical cartilage-removing techniques can complicate the auricular reconstruction and have not consistently demonstrated superior oncologic outcomes.^7^ Cartilage-sparing techniques, such as Mohs micrographic surgery (MMS), have gained prominence due to the enhanced ability to maintain form and function. Moreover, cartilage-sparing approaches, based on limited emerging data, may offer adequate or even superior oncological control compared to cartilage-removing techniques for local recurrence.^8^

The primary objective and aim of this study is to systematically review what is available in current literature on the differences in oncological outcome when performing a cartilage-sparing or a cartilage-removing excision of auricular melanoma. The results will guide decision making in the management of AM.

## METHODOLOGY

This is a systematic review adhering to PRISMA guidelines^8^ and following the PICOT (Population, Intervention, Comparison, Outcome, Time) framework^9^ to evaluate current evidence comparing oncological outcomes in cartilage-sparing and cartilage-removing excisions of auricular melanoma.

### Search

A systematic search was undertaken for all studies utilising cartilage-sparing and cartilage excising techniques for auricular melanoma. Articles were selected from the PubMed,^10^ Scopus,^11^ and MEDLINE^12^ databases. The MEDLINE search was performed using the OVID search engine.^13^ The authors conducted the search independently. Three groups of keywords were used: the first relating to melanoma, the second related to cartilage-sparing and cartilage excising techniques, and the third relating to the ear. The specific search terms and Boolean operators used for each database are detailed in Tables 1,2,3. All types of study were included in the search strategy. Following the search, all identified studies were uploaded to the latest version of the AI systematic review software, Rayyan^14^ and 2 authors (H.T. and M.C.) assessed the abstracts and evaluated the suitability for inclusion in the review; additionally, the bibliography list of each article was also screened to identify other relevant studies.

**TABLE 2:** Scopus Search Strategy

Date: March 20, 2025		
Number	Term	Results
1	“melanoma”	241,439
2	“cartilage-sparing” OR “cartilage sparing” OR “sparing of cartilage” OR “cartilage-preserving” OR “cartilage preserving” OR “preservation of cartilage” OR “cartilage preservation” OR “cartilage-retaining” OR “cartilage retaining” OR “retainment of cartilage” OR “cartilage-conserving” OR “cartilage conserving” OR “conservation of cartilage” OR “cartilage-harvested” OR “cartilage harvested” OR “cartilage-harvesting” OR “cartilage harvesting” OR “harvesting cartilage” OR “cartilage-excision” OR “cartilage excision” OR “excision of cartilage” OR “cartilage-resection” OR “cartilage resection” OR “resection of cartilage” OR “cartilage removal” OR “removal of cartilage” OR “chondrectomy” OR “chondrotomy”	1002
3	“ear” OR “auricular” OR “auricle” OR “pinna” OR “external ear” OR “middle ear” OR “inner ear” OR “helix” OR “antihelix” OR “lobule”	480,273
4	1 AND 2 AND 3	7

**TABLE 3:** MEDLINE Search Strategy

Date: March 20, 2025		
Number	Term	Results
1	“melanoma”.mp.	147,338
2	“cartilage-sparing”.mp. OR “cartilage sparing”.mp. OR “sparing of cartilage”.mp. OR “cartilage-preserving”.mp. OR “cartilage preserving”.mp. OR “preservation of cartilage”.mp. OR “cartilage preservation”.mp. OR “cartilage-retaining”.mp. OR “cartilage retaining”.mp. OR “retainment of cartilage”.mp. OR “cartilage-conserving”.mp. OR “cartilage conserving”.mp. OR “conservation of cartilage”.mp. OR “cartilage-harvested”.mp. OR “cartilage harvested”.mp. OR “cartilage-harvesting”.mp. OR “cartilage harvesting”.mp. OR “harvesting cartilage”.mp. OR “cartilage-excision”.mp. OR “cartilage excision”.mp. OR “excision of cartilage”.mp. OR “cartilage-resection”.mp. OR “cartilage resection”.mp. OR “resection of cartilage”.mp. OR “cartilage removal”.mp. OR “removal of cartilage”.mp. OR “chondrectomy”.mp. OR “chondrotomy”.mp.	730
3	“ear”.mp. OR “auricular”.mp. OR “auricle”.mp. OR “pinna”.mp. OR “external ear”.mp. OR “middle ear”.mp. OR “inner ear”.mp. OR “helix”.mp. OR “antihelix”.mp. OR “lobule”.mp.	247,808
4	1 AND 2 AND 3	3

**TABLE 4:** Study Characteristics

Study	Location	Design	Single- or Multicentered?	Sample	Age (y), Mean (Range)	Gender, n (%)	Follow-up (mo), Mean (Range)
Govaert et al,^20^ 2024	Belgium	Retrospective analysis	Single	14	66.6 (41–86)	M, 14 (100)	41 (15–132)
Yamasaki and Emerick,^21^ 2020	USA	Retrospective analysis	Single	8	53.5 (29–76)	M, 5 (63) F, 3 (38)	22.5 (3.6–46.2)
Truong et al,^22^ 2018	USA	Retrospective analysis	Single	156	62.5 (18–101)	M, 134 (85.9) F, 22 (14.1)	67.2 (0.48–186)
McCarty et al,^23^ 2013	USA	Retrospective analysis	Single	18	66.5 (42–82)	M, 16 (88.8) F, 2 (11.2)	30.5 (1–61)
Sartore et al,^17^ 2011	Italy	Retrospective analysis	Single	9	60.5 (37–75)	M, 8 (88.9) F, 1 (11.1)	76 (12–162)
Pockaj et al,^16^ 2003	USA	Retrospective analysis	Multi	78	64 (23–87)	M, 68 (87.2) F, 10 (12.8)	55.7 (0.4–139.8)

F, female; M, male; USA, United States of America.

### Selection

The study selection process was guided by the PICOT framework^9^:

- Population (P): patients diagnosed with auricular melanoma;
- Intervention (I): cartilage-sparing excision techniques;
- Comparison (C): cartilage-removing excision techniques;
- Outcome (O): recurrence and disease-specific mortality;
- Time (T): recurrence and disease-specific mortality assessed in both short-term (≤1 year) and long-term (≥1 year) follow-ups.

Eligible studies were limited to English only and were based on the excision of auricular melanoma in the context of cartilage-sparing and cartilage-excising techniques. Each study was required to clearly state the number of patients undergoing cartilage-sparing and/or cartilage-removing excision excisions of melanoma, have a minimum average follow-up of 12 months, and provide clear data on oncological outcomes. Additionally, correspondences or discussions to editors, case reports, literature reviews, or abstract-only publications were also excluded.

**FIGURE 2:**
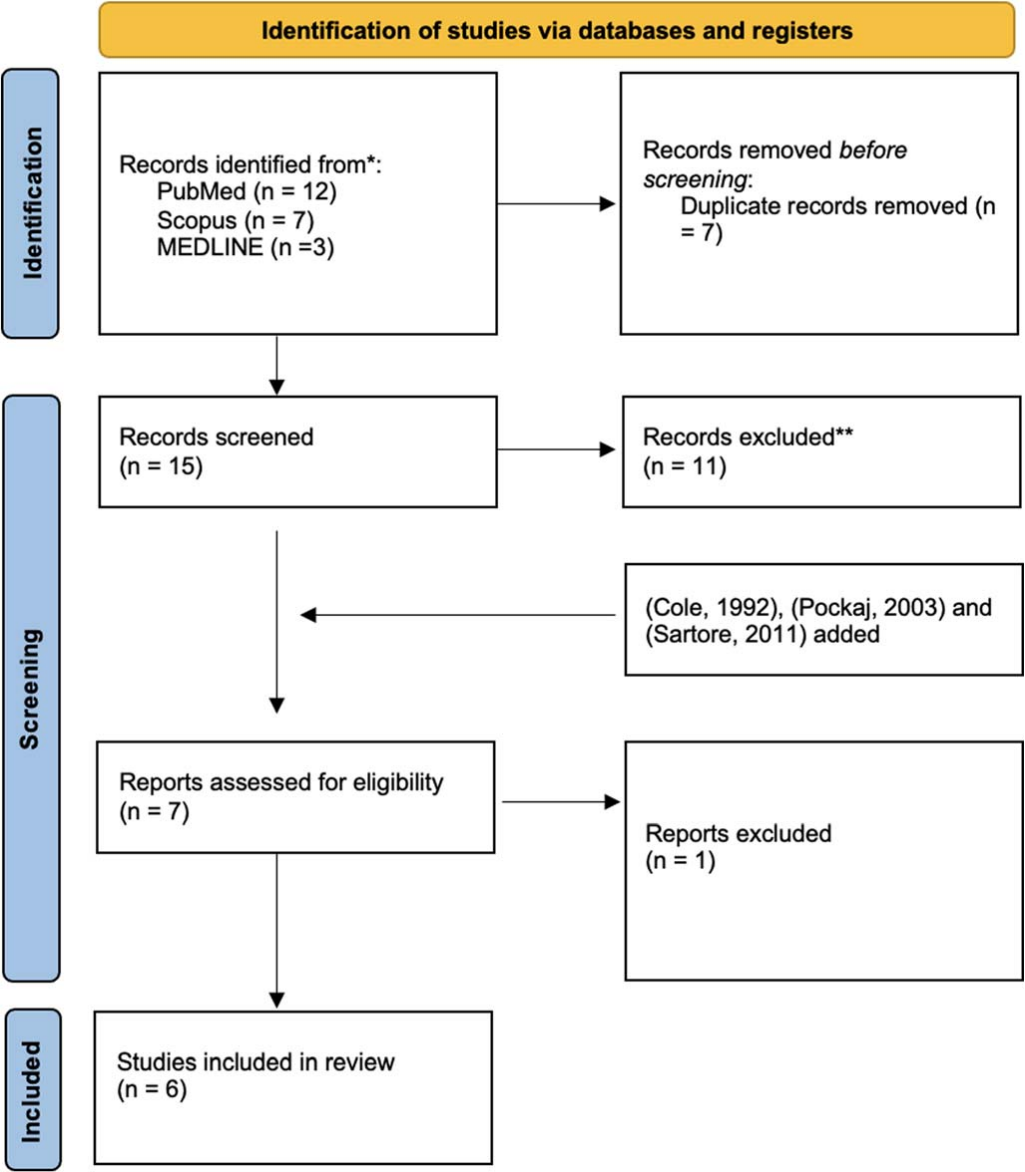
PRISMA flowchart.

The initial search identified 23 studies—12 from PubMed, 7 from Scopus, and 3 from MEDLINE. Following the removal of 7 duplicates, 15 studies remained to be independently screened by 2 of the authors. Of these, 11 were excluded for not meeting the inclusion criteria. During the screening process, 3 further articles on cartilage excision in auricular melanoma were identified through alternative means, although these were not captured by the initial search strategy for reasons that remain unclear.^15,16,17^ This left 7 studies that were quality assessed using the Risk of Bias in Nonrandomized Studies—of Interventions, Version 2 (ROBINS I-V2) assessment tool.^18^ Cole et al was excluded during the quality assessment stage due to the potential use of outdated surgical techniques.^15^ This left 6 studies that were included in the final review. Figure 2 visually summarises the study selection process.^19^

## RESULTS

The basic characteristics of each study including location, design, patient details, and length of follow-up period are detailed in Table 4. Characteristics of the auricular melanomas in each study are presented in Table 5. Details regarding the surgical approach used for melanoma excision, including the number of patients undergoing cartilage-sparing versus cartilage-removing techniques and associated clinical outcomes, are presented in Table 6. A direct comparison between cartilage-sparing and cartilage-removing techniques could not be performed through subanalysis. This limitation arose from the lack of clear stratification between the 2 groups in terms of patient characteristics, tumor features, and surgical methods across several studies. Consequently, the data are presented on a study-by-study basis.

**TABLE 5:** Melanoma Characteristics

Study	Histopathology, n (%)	Primary Melanoma Location, n (%)	Melanoma Stage at Diagnosis, n (%)	Breslow Thickness (mm), Mean/Median (Range)	Ulceration, n (%)	Lymphovascular Invasion, n (%)	Perineural Invasion, n (%)
Govaert et al,^20^ 2024	SSM, 7 (50) NM, 2 (14.3) LMM, 5 (35.7)	Helix, 9 (64.3) Antihelix, 3 (21.4) Lobule, 1 (7.1) Scapha, 1 (7.1)	pT1a, 6 (42.9) pT1b, 3 (21.4) pT2a, 1 (7.1) pT3a, 3 (21.4) pT3b, 1 (7.1)	1.24 (0.34–2.7)	Present, 2 (14.3) Absent, 10 (71.4) Unknown, 2 (14.3)	Present, 0 (0) Absent, 12 (85.7) Unknown, 2 (14.3)	Present, 0 (0) Absent, 12 (85.7) Unknown, 2 (14.3)
Yamasaki and Emerick,^21^ 2020	SSM, 4 (50) UC, 4 (50)	Helix, 6 (75) Postauricular, 1 (12.5) Triangular fossa, 1 (12.5)	pT1a, 1 (13) pT1b, 3 (38) pT2a, 2 (25) pT2b, 1 (13) pT3a, 1 (13)	1.18 (0.17–2.17)	Present, 1 (13) Absent, 7 (88)	Absent, 4 (50) Unknown, 4 (50)	Absent, 4 (50) Unknown, 4 (50)
Truong et al,^22^ 2018	SSM, 36 (23.1) NM, 13 (8.3) LMM, 56 (35.9) DP, 5 (3.3) Spitzoid, 3 (1.9) UC, 40 (25.6) Unknown, 3 (1.9)	Helix, 91 (58.3) Lobe, 20 (12.8) Other, 37 (23.7) Unknown, 8 (5.1)	IA, 55 (35.3) IB, 48 (30.8) IIA, 20 (12.8) IIB, 9 (5.8) Unknown, 24 (15.4)	0.86 (0.1–7)	Present, 29 (18.6) Absent, 115 (73.7) Unknown, 12 (7.7)	NR	NR
McCarty et al,^23^ 2013	SSM, 5 (27.8) NM, 5 (27.8) LMM, 7 (38.9) Unknown, 1 (5.6)	Helix, 7 (38.9) Lobule, 6 (33.3) Tragus, 3 (16.7) Concha, 1 (5.6) Crus of Helix, 1 (5.6)	Clark I, 3 (16.7) Clark II, 1 (5.6) Clark III, 3 (16.7) Clark IV, 10 (55.6) Unknown, 1 (5.6)	2.02 (0.4–6)	Present, 5 (27.8) Absent, 13 (72.2)	NR	NR
Sartore et al,^17^ 2011	SSM, 3 (33.3) NM, 2 (22.2) LMM, 2 (22.2) MNC, 1 (11.1) ISMF, 1 (11.1)	Helix, 4 (44.4) Lobe, 2 (22.2) Postauricular, 1 (11.1) Concha, 2 (22.2) EJS, 1 (11.1)	NR	2.10 (0.16–5.87)	Present, 1 (11.1) Absent, 8 (88.9)	NR	NR
Pockaj et al,^16^ 2003	NR	Helix, 50 (64.1) Lobule, 13 (16.7) Scapha, 1 (1.3) Concha, 5 (6.4) Tragus, 3 (3.8) Unknown, 6 (7.7)	NR	1.7 (0.2–7)	NR	NR	NR

DP, desmoplastic; EJS, ear junction-scalp; ISMF, in situ melanoma with focal infiltration; LMM, lentigo maligna melanoma; MNC, melanoma with nevoid characteristics; NM, nodular melanoma; NR, not reported; SSM, superficial spreading melanoma; UC, unclassified.

**TABLE 6:** Details on Surgical Procedure and Clinical Outcomes

Study	Surgical Procedure, n (%)	Radial Resection Margins (cm), Range	Reconstruction Method, n (%)	Cartilage Spared, n (%)	Cartilage Removed, n (%)	Sentinel Lymph Node Biopsy (SLNB) Status, n (%)	Recurrence, n (%)	Time Until Recurrence (mo), Mean	Disease-Specific Mortality, n (%)
Govaert et al,^20^ 2024	WLE*, 14 (100)	0.6–5	PC, 1 (7.1) STSG, 9 (63.9) FTSG, 4 (28.6)	14 (100)	0 (0)	Positive, 0 (0) Negative, 14 (100) Unknown, 0 (0)	Total, 2 (14.3) Local, 1 (7.1) Locoregional, 1 (7.1) Distant, 0 (0)	117.55	0 (0)
Yamasaki and Emerick,^21^ 2020	WLE, 8 (100)	1–2	FTSG, 8 (100)	8 (100)	0 (0)	Positive, 1 (14) Negative, 6 (86) Unknown, 1 (14)	Total, 1 (14) Locoregional, 1 (14)	33.6	0 (0)
Truong et al,^22^ 2018	WLE, 14 (9) WR, 101 (64.7) AURICX, 14 (9) SE, 17 (10.9) MS, 2 (1.3) Unknown, 8 (5.1)	N/A	NR	29 (18.6)	122 (78.2)	Positive, 14 (9) Negative, 85 (54.4) Unknown, 57 (36.5)	Total, 26 (16.7) Local, 4 (2.6) Regional, 5 (3.2) Distant, 10 (6.4) Locoregional, 1 (0.6) LD, 2 (1.3) RD, 3 (1.9) Unknown, 1 (0.6)	27.6	7 (4.5)
McCarty et al,^23^ 2013	WLE, 18 (100)	1–2	STSG, 2 (11.1) FTSG, 14 (77.8) FTSG + AF, 1 (5.6) FTSG + TF, 1 (5.6)	18 (100)	0 (0)	Positive, 1 (5.6) Negative, 6 (33.3) Unknown, 11 (61.1)	Total, 4 (22.2) Locoregional, 2 (11.1) Distant, 2 (11.1)	33	2 (11.1)
Sartore et al,^17^ 2011	WLE, 5 (55.6) P-AURICX, 2 (22.2) Unknown, 2 (22.2)	0.5–1.5	PC, 1 (11.1) FTSG, 1 (11.1) CCAF, 5 (55.6) N/A, 2 (22.2)	7 (77.8)	2 (22.2)	Negative, 5 (55.6) Unknown, 4 (44.4)	Total, 1 (11.1) Local, 1 (11.1)	38	1 (11.1)
Pockaj et al,^16^ 2003	WR, 45 (57.7) AURICX, 5 (6.4) P-AURICX, 8 (10.3) MS, 11 (14.1) SPC, 7 (8.9) Unknown, 2 (2.6)	N/A	NR	7 (8.9)	71 (91.1)	Negative, 10 (12.8) Unknown, 68 (87.2)	Total, 19 (24.4) Local, 10 (12.8) Regional, 9 (11.5) Distant, 17 (21.8)	NR	18 (23.1)

AF, advancement flap; AURICX, auriculectomy; CCAF, chondrocutaneous advancement flap; FTSG, full-thickness skin graft; LD, local + distant; MS, Mohs surgery; N/A, not applicable; NR, not reported; P-AURICX, partial auriculectomy; PC, primary closure; RD, regional + distant; SE, staged excision; SPC, skin, with preservation of the perichondrium; STSG, split thickness skin graft; TF, transposition flap; WLE, wide local excision; WR, wedge resection.

### Study Characteristics

A total of 6 studies were included in the final review, all of which adopted a retrospective analysis approach.^16,17,20,21,22,23^ Of these, Pockaj et al was the only multicenter study.^16^ Sample sizes ranged from 8 to 156 patients with a median of 16.^21,22^ The cumulative average age of participants across the review was 62.3 years (range, 53.5–66.6 years). All studies reported a predominance of male patients. The cumulative average follow-up period was 48.8 months, ranging from 30.5 to 76 months.

#### Histopathological Subtype

Govaert et al, McCarty et al, and Sartore et al identified superficial spreading malignant melanoma as the most common histopathological subtype within their patient populations.^17,20,23^ In contrast, Truong et al reported unclassified melanoma as the most frequently observed subtype, whereas Yamasaki and Emerick reported an equal distribution between superficial spreading and nodular malignant melanoma.^21,22^ Pockaj et al was the only study to not report adequate data on the histopathological subtypes present in its cohort.^16^

#### Location of Primary Melanoma

All studies reported adequate data on the anatomical location of the primary auricular melanoma in their patient populations. Across the review, the auricular helix was identified as the most common site of tumor origin.

### Breslow Thickness

All included studies reported Breslow thickness for their respective patient populations. Most studies provided the mean Breslow thickness, except for Truong et al, which reported the median value of 0.86 mm (range, 0.1–7.0 mm).^22^ Among the studies that reported mean values, the cumulative average Breslow thickness was 1.65 mm (range, 1.18–2.10 mm). Sartore et al documented the highest mean Breslow thickness at 2.10 mm (range, 0.16–5.87 mm),^17^ whereas Yamasaki and Emerick reported the lowest at 1.18 mm (range, 0.17–2.17 mm).^21^

### Ulceration, Lymphovascular, and Perineural Invasion

Ulceration was absent in ≥70% of patients in all studies that reported relevant data.^17,20,21,22,23^ Pockaj et al did not report information regarding ulceration status.^16^ Reporting on lymphovascular and perineural invasion was limited across the reviewed studies, with only Govaert et al and Yamasaki and Emerick presenting sufficient data.^20,21^ Govaert et al reported the absence of lymphovascular and perineural invasion in 12 patients (85.7%) with the status unknown in 2 patients (14.3%).^20^ Similarly, Yamasaki and Emerick found no lymphovascular or perineural invasion in 4 patients (50%), whereas the status was unknown in the remaining 4 patients (50%).^21^

#### Surgical Procedure, Excision Margins, and Method of Reconstruction

Wide local excision (WLE) was identified as the most frequently utilised technique for the removal of auricular melanoma. Govaert et al (n = 14), Yamasaki and Emerick (n = 8), and McCarty et al (n = 18) reported exclusive use of WLE in their respective cohorts.^20,21,23^ Sartore et al primarily utilised WLE (n = 5) but also performed partial auriculectomy (n = 2), with the surgical approach undocumented in a further 2 cases.^17^ The largest study, Truong et al, most frequently employed wedge resection (n = 101) but also reported the use of WLE (n = 14), total auriculectomy (n = 14), staged excision (n = 17), and Mohs surgery (n = 2), with 8 further patients having an unidentifiable surgical technique.^22^ Similarly, Pockaj et al predominantly used wedge resection (n = 45), alongside partial auriculectomy (n = 8), total auriculectomy (n = 5), Mohs surgery (n = 11), and excision with preservation of the perichondrium (n = 7), with the technique being unknown in 2 cases.^16^ Of the studies that reported data on excision margins, the cumulative range was 0.5 to 5 cm.^17,20,21,23^ Excision margins were not reported in Truong et al or Pockaj et al.^16,22^

A range of reconstruction methods were reported across the review. Govaert et al most frequently utilised split-thickness skin grafts (STSGs; n = 9) but also reported use of full-thickness skin grafts (FTSGs; n = 4) and primary closure (PC; n = 1).^20^ Yamasaki and Emerick exclusively used FTSG for all patients (n = 8).^21^ McCarty et al primarily employed FTSG (n = 14), with additional use of FTSG in combination with advancement (n = 1) and transposition flaps (n = 1), and STSG in 2 patients.^23^ Sartore et al most commonly utilised chondrocutaneous advancement flaps (n = 5) but also used PC (n = 1) and FTSG (n = 1); reconstruction modality was unspecified in 2 patients.^17^ Neither Truong et al nor Pockaj et al reported details on the methods of auricular reconstruction within their patient populations.^16,22^

#### Proportion of Patients Undergoing Cartilage-Sparing and Cartilage-Removing Excisions

Govaert et al (n = 14), Yamasaki and Emerick (n = 8), and McCarty et al (n = 18) exclusively spared the auricular cartilage in their patient populations.^20,21,23^ In contrast, Truong et al reported cartilage preservation in 29 patients (18.6%) and removal in 122 patients (78.2%), with cartilage status unidentifiable in 5 (3.3%) patients.^22^ Sartore et al preserved cartilage in 7 patients (77.8%) and excised it in 1 patient (22.2%).^17^ Finally, Pockaj et al retained the cartilage of 7 patients (8.9%) and removed it in 71 patients (91.1%).^16^

#### Recurrence

Recurrence rates were adequately reported in all studies. The overall recurrence rate ranged from 11.1% to 24.4%. Specifically, local recurrence ranged from 2.6% to 12.8%, regional recurrence ranged from 3.2% to 11.5%, and distant metastasis ranged from 0 to 21.8%. Although some studies reported additional classifications such as locoregional or combined local and distant recurrence, only those that explicitly specified local, regional, or distant recurrence were included in the ranges above. Excluding Pockaj et al due to a lack of reporting, the average time to recurrence across the remaining studies was calculated to be 33.6 months (range, 27.6–117.6 months).^16^ Truong et al reported the highest absolute number of patients experiencing recurrence, with 26 patients (16.7%). Among these, regional (n = 5) and distant metastasis (n = 10) were the most common patterns of recurrence, with a mean time to recurrence of 27.6 months. Notably, auricular cartilage was excised in 20 of these patients (76.9%), preserved in 3 (11.5%), and was of unknown status in the remaining 3 (11.5%).^22^

Pockaj et al reported the highest proportion of recurrence, affecting 19 of 78 patients (24.4%). Of these, 10 developed local recurrence, 9 had regional recurrence, and 17 developed distant metastases. Auricular cartilage had been removed in 17 of the 19 patients (21.8%) who experienced recurrence. Cartilage was spared in the remaining 2 patients (2.6%) with 1 undergoing Mohs surgery, and the other had only a diagnostic biopsy; the average time to recurrence was not reported.^16^ Yamasaki and Emerick and Sartore et al reported the lowest number of recurrences, with 1 patient each (14% and 11.1% of their respective cohorts). The mean time to recurrence was 33.6 months and 38 months, respectively. In Yamasaki and Emerick, cartilage was preserved in all patients. Notably, the sole patient who experienced recurrence had the narrowest deep margin in the cohort.^21^ In Sartore et al, the patient who developed recurrence underwent a partial auriculectomy; therefore, it is presumed that cartilage excision was performed. This patient also demonstrated a substantial Breslow thickness of 4.5 mm and was the only patient in the study with ulceration present, which likely contributed to the recurrence.^17^

#### Disease-Specific Mortality

Disease-specific mortality was consistently reported across the included studies. Pockaj et al documented the highest disease-specific mortality, with 18 patients (23.1%) succumbing to auricular melanoma-related causes.^16^ In Truong et al, 7 patients (4.5%) died as a direct result of the disease.^22^ McCarty et al and Sartore et al reported disease-specific mortality in 2 patients (11.1%) and 1 patient (11.1%), respectively.^17,23^ No disease-related deaths were observed during the follow-up periods reported by Govaert et al and Yamasaki and Emerick.^20,21^ Although the differences in average Breslow thickness among the studies were relatively small, those with a higher upper limit were associated with increased disease-specific mortality. For instance, the upper limit of Breslow thickness reported by both Pockaj et al and Truong et al was 7 mm, whereas it was notably lower in Govaert et al and Yamasaki and Emerick, at 2.7 mm and 2.17 mm, respectively.^16,20,21,22^

## DISCUSSION

This systematic review aimed to explore what is currently available in medical literature on the oncological outcomes of cartilage-sparing and cartilage-removing excisions of auricular melanoma. Several key observations can be made following the review and subsequent evaluation of patient data; however, these findings are undermined by several limitations.

Truong et al and Pockaj et al predominantly included patient cohorts in which the auricular cartilage was excised alongside the melanoma. Notably, these 2 studies also reported the highest recurrence rates among all included studies, excluding McCarty et al, where most patients had auricular melanomas graded as Clark level IV. Whilst Pockaj et al did not report the time to recurrence, Truong et al reported the shortest average time to recurrence (27.6 months), compared to studies that primarily employed cartilage-sparing approaches. Furthermore, both studies demonstrated that the majority of patients who developed recurrence had undergone cartilage-removing surgery: 76.9% in Truong et al and 100% in Pockaj et al. These findings are particularly noteworthy given that Truong et al reported a low median Breslow thickness (0.86 mm) and the majority of patients who experienced recurrence had nonulcerated primary lesions (77.3%). This raises important questions regarding the etiology of recurrence in this context. Additionally, Pockaj et al reported the highest disease-specific mortality rate (23.1%), further suggesting a potential association between cartilage excision and poorer oncological outcomes. Collectively, these observations warrant further investigation into whether cartilage preservation may alter disease pathogenesis resulting in improved disease control and survival in patients with AM.

Govaert et al, Yamasaki and Emerick, and Sartore et al all adopted cartilage-preserving surgical techniques in the management of auricular melanoma. Whilst Sartore et al did not report the staging of melanoma at diagnosis, the majority of patients in both Govaert et al and Yamasaki and Emerick were classified as pT1a, pT1b, or pT2a. These 3 studies demonstrated lower absolute numbers and proportions of patients experiencing recurrence, as well as longer average times to recurrence, when compared to studies that involved cartilage excision. These findings raise the possibility that preservation of the auricular perichondrium may offer a protective barrier against disease recurrence. This hypothesis is further supported by the consistently lower recurrence rates and disease-specific mortality observed in these cohorts.

In contrast, McCarty et al exclusively employed cartilage-sparing excisions but reported the highest proportion of patients experiencing recurrence and disease-specific mortality among the cartilage-preserving studies. This study reported the second-highest mean Breslow thickness across the review (2.02 mm), surpassed only by Sartore et al. Furthermore, McCarty et al demonstrated the highest rate of ulceration, which was present in 27.8% of patients. These findings markedly differ from those of Govaert et al and Yamasaki and Emerick, which also utilised cartilage-sparing approaches but reported lower recurrence rates. This discrepancy is likely attributable to the fact that Govaert et al and Yamasaki and Emerick were consisted of patients with predominantly lower-stage melanomas, reduced Breslow thickness, and less presence of ulceration. Collectively, the literature suggests that cartilage-sparing excisions may be oncologically safe in cases of auricular melanoma that present with early TNM stage, low Breslow thickness, and absence of ulceration.

In comparison to another review on the topic, Harrison et al conducted a literature review alongside a retrospective analysis of patients treated with either cartilage-sparing or cartilage-excising techniques for auricular melanoma at their center.^24^ This study concluded no significant difference in recurrence rates between cartilage-sparing and cartilage-excising approaches. This finding stands in contrast to the conclusions of this review. It is important to note, however, that Harrison et al did not include the data from some studies in this review.^20,21,22^ As such, the existing evidence base has evolved, highlighting the need for an updated systematic review incorporating these more recent studies.

There are several limitations that undermine the findings of this review. It is important to consider that a fundamental selection bias may influence the reported outcomes in the included studies. Cartilage-removing procedures are often utilised for more advanced-stage tumors, where perichondral involvement is suspected or confirmed. As a result, the poorer oncological outcomes may reflect the aggressive nature of melanoma rather than the efficacy of the surgical technique itself. This makes it difficult to draw definitive conclusions regarding the relative effectiveness of cartilage-sparing versus cartilage-removing approaches and undermines the generalisability of this review. Future studies that stratify outcomes by tumor stage and extent of cartilage involvement are needed to isolate the independent effect of surgical technique on oncological outcomes.

In studies that included patients treated with both cartilage-sparing and cartilage-excising approaches, recurrence data were adequately reported, but patient and tumor characteristics were not always differentiated between the 2 surgical groups. As a result, data within this review had to be presented in a study-by-study format, rather than enabling direct comparisons or subgroup analyses between cartilage-sparing and cartilage-excising cohorts. This limitation impacts several domains of the review. External validity is reduced, as the inability to perform subgroup analysis restricts the capacity to determine which surgical technique may offer superior oncological outcomes. Furthermore, the lack of stratification in prognostic factors such as Breslow thickness, tumor location, and patient demographics introduces potential confounding variables, which may have influenced recurrence or survival outcomes independently of the surgical method employed. This weakens the internal validity of the review and limits the ability to draw conclusive inferences between the type of surgical excision and recurrence or mortality outcomes. To address these limitations, future studies should ensure comprehensive and consistent reporting of tumor and patient characteristics, clearly stratified by surgical technique, to allow for more rigorous comparative analyses.

The internal validity of the review is further limited by the absence of randomised controlled trials (RCTs) and the consequent reliance on retrospective analyses, which are inherently more susceptible to bias. The reliability, accuracy, and generalisability of the findings are further compromised by the small median sample size of 16 (range, 8–156), as conclusions drawn from limited cohorts are unlikely to be representative of the broader population. Inconsistencies in reporting across the included studies further undermine their methodologies. Additionally, the omission of key histopathological features such as regression, perineural invasion, and lymphovascular invasion diminishes this review's prognostic accuracy, as these are well-established indicators of recurrence risk. Furthermore, the absence of data on postoperative complications across all studies limits the external validity, restricting the applicability of findings to clinical practice, particularly in evaluating the relative safety of different surgical approaches.

A further hypothesis could be made regarding auricular cartilage's role as a natural anatomical barrier and whether this may have a protective effect against spreading melanoma. Cartilage may act as a partial physical barrier that limits intersurface tumor spread; therefore, its excision could hypothetically facilitate transcartilaginous dissemination, potentially exposing tumor cells to a broader lymphatic network. This could contribute to the increased recurrence and metastasis rates observed in some cartilage-removing cohorts. Although direct histopathological or clinical evidence supporting this mechanism is lacking, it presents an underexplored avenue for future research and may partially explain discrepancies in oncological outcomes between surgical techniques.

Further research is necessary to enable a robust comparison of the oncological sequelae associated with cartilage-sparing versus cartilage-excising surgical approaches in the management of auricular melanoma. Ideally, future investigations should involve long-term randomised controlled trials with clear stratification between patients undergoing cartilage-sparing and cartilage-excising procedures.

## CONCLUSIONS

In conclusion, although several studies included in this review are limited by methodological factors such as suboptimal study design, small sample sizes, inadequate stratification of patient populations, and inconsistent reporting of melanoma characteristics, the central hypothesis is partially supported. Evidence suggests a potential difference in recurrence rates between cartilage-sparing and cartilage-removing approaches to auricular melanoma excision. This is inferred from notable disparities in recurrence rates and disease-specific mortality between studies exclusively employing cartilage-sparing techniques and those including a significant proportion of cartilage-removing procedures. However, the underlying mechanisms, direction, and magnitude of this observed difference remain unclear due to the paucity of high-quality data and the frequent failure to clearly distinguish between surgical approaches within individual studies. Further limitations stem from the reliance on non-randomised designs, which hinder accurate cross-study comparisons. To establish more definitive conclusions regarding oncological outcomes associated with cartilage-sparing versus cartilage-removing excisions, well-designed, long-term randomised controlled trials are needed.
